# The Affective Reactivity Index: a concise irritability scale for clinical and research settings

**DOI:** 10.1111/j.1469-7610.2012.02561.x

**Published:** 2012-11

**Authors:** Argyris Stringaris, Robert Goodman, Sumudu Ferdinando, Varun Razdan, Eli Muhrer, Ellen Leibenluft, Melissa A Brotman

**Affiliations:** 1 King’s College London, Institute of PsychiatryDenmark Hill, London, UK; 2 Section on Bipolar Spectrum Disorders, Emotion and Development Branch, National Institute of Mental Health, National Institutes of Health, Department of Health and Human ServicesBethesda, MD USA

**Keywords:** Mood dysregulation, Affective Reactivity Index, irritability, depression, bipolar

## Abstract

**Background:**

Irritable mood has recently become a matter of intense scientific interest. Here, we present data from two samples, one from the United States and the other from the United Kingdom, demonstrating the clinical and research utility of the parent- and self-report forms of the Affective Reactivity Index (ARI), a concise dimensional measure of irritability.

**Methods:**

The US sample (*n* = 218) consisted of children and adolescents recruited at the National Institute of Mental Health meeting criteria for bipolar disorder (BD, *n* = 39), severe mood dysregulation (SMD, *n* = 67), children at family risk for BD (*n* = 35), or were healthy volunteers (*n* = 77). The UK sample (*n* = 88) was comprised of children from a generic mental health setting and healthy volunteers from primary and secondary schools.

**Results:**

Parent- and self-report scales of the ARI showed excellent internal consistencies and formed a single factor in the two samples. In the US sample, the ARI showed a gradation with irritability significantly increasing from healthy volunteers through to SMD. Irritability was significantly higher in SMD than in BD by parent-report, but this did not reach significance by self-report. In the UK sample, parent-rated irritability was differentially related to emotional problems.

**Conclusions:**

Irritability can be measured using a concise instrument both in a highly specialized US, as well as a general UK child mental health setting.

## Introduction

Recently, irritable mood has become a focus of intense scientific interest (Leibenluft, 2011; Stringaris, 2011). However, research on the measurement of irritability has been limited. This study reports the psychometric properties of a concise irritability measure for use in clinical practice and research.

While irritability is listed as a symptom for multiple diagnoses, the term is not defined in the DSM-IV (APA, 2000), and there is no consensus definition in the literature. Moreover, despite the intense interest of the DSM-5 taskforce in irritability, defined both dimensionally and categorically (APA, 2011a,b), research on the measurement of irritability has been limited. This is unfortunate, given the importance ascribed to studying the dimensional structure of psychopathology and its neurobiological underpinnings (Insel et al., 2010).

To address this gap, we developed the Affective Reactivity Index (ARI^1^), a scale that contains six symptom items and one impairment item about irritability. We chose the item contents based on a simple, broad definition of irritability as a mood of easy annoyance and touchiness characterized by anger and temper outbursts (Stringaris, 2011). Respondents rate irritability over the last 6 months. The scale focuses on chronic irritability (Leibenluft, Cohen, Gorrindo, Brook, & Pine, 2006). This presents commonly as a child who is described by his/her parents as ‘always angry’ and as reacting with intense anger to situations that other children would take in their stride. This differs from the less usual presentation of irritability occurring as part of a circumscribed episode (APA, 2000; Leibenluft et al., 2006).

Specifically, the ARI scale was designed to examine, in a way accessible to most children and parents, three aspects of irritability: (a) threshold for an angry reaction; (b) frequency of angry feelings/behaviors; (c) duration of such feelings/behaviors.

The scale was designed to ascertain irritable mood rather than its possible consequences such as hostility [i.e., dislike toward particular people (Buss & Durkee, 1957)], or acts of aggression (e.g., hitting others or damaging property). Aggression and hostility may or may not occur with irritability. For example, irritability may be observable to the parent as the non-aggressive ‘huffing and puffing’ of a child whose wish has been thwarted. To the child, irritable mood may be present as a feeling that does not necessarily motivate aggressive action. Previous scales measuring irritability or trait anger frequently contain items of aggressive, antisocial, or hyperactive behavior and symptoms, such as ‘non-profitable damage to property’ (Vitiello, Behar, Hunt, Stoff, & Ricciuti, 1990), ‘I feel I might lose control and hit or hurt someone’ (Snaith & Taylor, 1985), ‘I attack whatever makes me angry’ (Jacobs, Phelps, & Rohrs, 1989), ‘pick fights with anyone’, ‘just can’t sit still’ (Mckinnie Burney, 2001), and ‘shout, kick, hit, let off steam’ (Caprara et al., 1985).

In addition, the ARI was specifically designed to obtain comparable information from youth *and* their parents. Some existing scales are available for adolescent informants only (Del Barrio, Aluja, & Spielberger, 2004), which is a limitation when doing research in developmental psychiatry (Angold, 2002). Finally, the scale was specifically designed to be (a) concise, which is in contrast to some of the existing scales (Buss & Durkee, 1957; Del Barrio et al., 2004; Mckinnie Burney, 2001); (b) as simple as possible [e.g., avoid complex items such as ‘I feel infuriated when I do a good job and get poor evaluation’ (Del Barrio et al., 2004) or ‘People pretend they are telling the truth, when they are really telling lies’ (Novaco & Taylor, 2004)]; (c) suitable for use as a screening instrument in busy clinics and epidemiologic studies.

This article reports the properties of the ARI in a US- and a UK-sample. The first aim of this study was to report item-level descriptive statistics. In this aim, we also sought to examine the internal consistencies and test that a single-factor structure is appropriate in the two samples. As part of the first aim, we also present preliminary data on the longitudinal stability of the scale and compare the scale’s properties across a US and a UK sample. The second aim is to test the association of the scale with psychopathology, using two approaches. The first, undertaken in the US sample, compares four groups: healthy volunteers; unaffected children at family risk for BD, that is, those with a first degree relative diagnosed with bipolar disorder (BD); children with BD; and children with severe mood dysregulation [SMD;(Leibenluft, 2011)]. Consistent with a dimensional view of irritability, we expect a graded increase of irritability from healthy volunteers through children at family risk for bipolar disorder and BD to SMD. We also test the hypothesis that the scale would distinguish between a group of patients selected for irritability, that is, those with SMD, compared with patients with other severe psychopathology, such as BD. This is important given the debate concerning the diagnostic boundaries of BD in youth. It had been claimed that severe irritability, even when it is not part of distinct episodes of altered mood, should be considered a hallmark of pediatric BD (Spencer et al., 2001; Wozniak et al., 1995). However, research on the SMD syndrome (Leibenluft, 2011; Leibenluft, Charney, Towbin, Bhangoo, & Pine, 2003), which is characterized by non-episodic severe irritability, suggests that SMD is unlikely to progress to BD (Brotman et al., 2006; Stringaris et al., 2010), does not share family risk with BD (Brotman et al., 2007), and has neural substrates separable from BD (Brotman et al., 2010).

Our second approach to testing the association of the scale with psychopathology is applied to the UK sample. We test the hypothesis that irritability will be differentially associated with emotional, rather than conduct or hyperactivity, symptoms. Theoretical considerations (Burke, Loeber, Lahey, & Rathouz, 2005; Wakschlag, Tolan, & Leventhal, 2010) underlie this hypothesis, as well as a host of recent evidence, that irritability shows stronger associations with emotional problems, rather than conduct problems or antisocial behaviors (Aebi et al., 2010; Rowe, Costello, Angold, Copeland, & Maughan, 2010; Stringaris, Cohen, Pine, & Leibenluft, 2009; Stringaris & Goodman, 2009a,b). The scales used for this previous research were generated ad hoc using items from existing instruments, rather than ones specifically designed, to measure irritability – these contained only a few items and had low internal consistency (Aebi et al., 2010; Stringaris & Goodman, 2009a,b). Here, we test the hypothesis that irritability will remain associated with emotional problems – but not with conduct problems – when controlling for other variables such as hyperactivity, peer problems, or prosocial behaviors.

## Methods

### The Affective Reactivity Index

The ARI was created as a parent- and a self-rated measure. Parents are presented with the following instruction sentence: ‘In the *last 6 months* and compared to others of the same age, how well does each of the following statements describe the behavior/feelings of your child? Please try to answer all questions.’ The self-report version is identical apart from referring to ‘*your* behaviour/feelings’). After the introduction, respondents are presented with six items related to feelings/behaviors specific for irritability (see Table 1), and one question assessing impairment due to irritability (‘overall, irritability causes him/her (or “me” by self-report) problems’). Each item has a three-level response category: ‘not true’, ‘somewhat true’, ‘certainly true’– scored as ‘0’, ‘1’, ‘2’, respectively, giving a range of possible scores of 0–12. Identical items comprise the parent- and self-report scales. The total score is the sum of the first six items. The impairment item is not counted in the total score. The questionnaire was derived from a longer (21-item) version, designed to contain redundancies. After piloting on 80 US cases and controls, it was reduced according to aims about coverage of duration, frequency, threshold (see Introduction), and parsimony (items that did not improve internal consistency or discrimination between cases and controls were dropped). The ARI scales are copyrighted and available without charge from the first author.

**Table 1:** Mean scores and factor loadings for the ARI items across reporting source in the two samples

	US sample				UK sample			
	Mean (*SD*) *parent n* = 214	Mean (*SD*) *self n* = 194	Factor Score *parent*	Factor Score *self*	Mean (*SD*) *parent n* = 83	Mean (*SD*) *self n* = 50	Factor Score *parent*	Factor Score *self*
Easily annoyed by others	0.86 (0.82)	0.87 (0.70)	0.88	0.77	0.84 (0.77)	0.74 (0.69)	0.68	0.90
Often lose temper	0.72 (0.84)	0.62 (0.70)	0.96	0.91	0.69 (0.80)	0.62 (0.75)	0.97	0.96
Stay angry for a long time	0.38 (0.58)	0.37 (0.58)	0.81	0.72	0.49 (0.72)	0.52 (0.71)	0.81	0.78
Angry most of the time	0.29 (0.59)	0.18 (0.45)	0.89	0.81	0.16 (0.40)	0.30 (0.58)	0.82	0.88
Get angry frequently	0.63 (0.82)	0.48 (0.69)	0.97	0.92	0.51 (0.72)	0.46 (0.71)	0.97	0.94
Lose temper easily	0.76 (0.88)	0.61 (0.78)	0.97	0.98	0.65 (0.72)	0.68 (0.79)	0.97	0.93
CFI			0.99	1.00			1.00	0.99
TLI			1.00	1.00			1.00	0.98
RMSEA			0.05	0.09			0.00	0.21
WRMR			0.42	0.60			0.38	0.84

CFI, comparative fit index; RMSEA, root mean square error of approximation; TLI, Tucker Lewis Index; WRMR, weighted root mean square residual.

### US sample

This sample is part of an ongoing study at the National Institute of Mental Health (NIMH), which has been previously described (Brotman et al., 2007; Stringaris et al., 2010). Here, we present data on those patients who completed the ARI from March 2009 (when it was introduced) through August 2011. Patients with SMD, or Bipolar Disorder Type I or Type II (BD), as well as children at family risk for BD were recruited through advertisements in support groups and with local psychiatrists, healthy volunteers were recruited through advertisements. Details about the diagnoses of BD and SMD can be found in the Appendix S1.

There were 218 participants in the US sample of whom 214 (98%) had ARI parent data, 194 (89%) had self-report data, and 192 (88%) had data by both reporting sources. The sample mean age was 12.90 years (*SD* = 2.70; range 6–17) with 130 (60%) boys. Diagnoses were: 67 (31%) with SMD, 39 (18%) with BD, 35 (16%) children at family risk for BD (i.e., first-degree BD relative), and 77 (35%) healthy volunteers. Data on comorbid diagnoses were available in all cases except: two cases with SMD, one case with BD, and two cases of children at family risk for BD. Of those with data on comorbidity, ADHD was also present in 55/65 (85%) of those with SMD and 32/38 (84%) of those with BD, while ODD was present in 54/65 (83%) of those with SMD, and 16/38 (42%) of those with BD. Of the BD subjects, 25/38 (66%) of the BD patients were euthymic at assessment, while 11/38 (29%) were hypomanic, 1/38 (3%) depressed, and 1/38 (3%) mixed. Further details about comorbidity and mood state can be found in the Appendix S1.

### Assessment of the US sample

In addition to the measurement of irritability using the ARI, the Kiddie Schedule for Affective Disorders – Present and Lifetime Version (KSADS-PL) (Kaufman et al., 1997) was administered to parents and children separately by clinicians with graduate level training and established reliability (*κ* = 0.9, including differentiating SMD and BD). Diagnoses were based on best-estimate procedures (Leckman, Sholomskas, Thompson, Belanger, & Weissman, 1982), generated in a consensus conference led by at least one psychiatrist with extensive experience evaluating children with bipolar-spectrum illness. SMD was assessed using a KSADS supplementary module (Leibenluft et al., 2003). Diagnoses in the relatives of children at family risk for BD were confirmed by KSADS-PL(Kaufman et al., 1997) for child siblings with BD or, for parents or adult siblings with BD, the Structured Clinical Interview for DSM-IV-TR Axis I Disorders-Patient Edition (SCID-I/P) (First, Spitzer, Gibbon, & Williams, 2002) or the Diagnostic Interview for Genetic Studies (DIGS) (Nurnberger et al., 1994). KSADS-PL was also used to determine diagnoses in the children at family risk for BD.

### Repeated ARI assessments

A small fraction of participants (*n* = 19 by parent-report; *n* = 11 by self-report) completed the ARI twice as part of ongoing follow-up (*M* 1.12 years, *SD* 0.36). For the participants who completed the ARI twice, we used only the Time 1 data in the analyses described in the rest of this article.

### UK sample

The *clinic sample* (*n* = 34) consisted of patients, aged 5–17 years, referred to the Community Child & Adolescent Mental Health Services of the South West London & St Georges Mental Health NHS Trust. Participation in the study was offered by the Specialist Registrar at the Service (co-author SF) to the patients allocated to her. The most common diagnoses in the clinic sample were: ODD (15%, *n* = 5), ADHD (15%, *n* = 5), autism spectrum disorder (ASD, 9%, *n* = 3). Also, 9% (*n* = 3) of cases presented with self-harm without a definite diagnosis. Only the primary diagnosis provided by the Registrar was used in this study. Further details of the sample are described in the Appendix S1.

The *control sample* (*n* = 54), consisting of participants aged 6–18 years, was recruited from one primary school and three secondary schools belonging to the same geographical area as the clinic. The head teacher in each school was approached and written informed consent was obtained for their school to participate in the study. The head teacher then invited potential study participants by handing out the questionnaires and consent forms to parents and young people in their school. The questionnaires were handed to those families who were more likely to return completed questionnaires as judged by the head teacher. The response rate from the primary school was 80%, while the average across the three secondary schools was 30%. Students with severe intellectual disability were excluded. The control sample was assumed to have no psychiatric diagnosis.

The mean age of the overall sample was 11.70 (*SD* = 3.46, range 5–18) with 59% (*n* = 52) boys. There were no significant differences between the clinic and the community sample with regard to age and gender (see Appendix S1).

*ARI completion rates*: of the 88 UK participants, 83 (94%) had ARI parent data. Self-reported ARI was only collected from children aged 11 and above: 52 children in this sample were 11 years of age or older and self-report ARI data were available on 50 (96%) of them, while 45 (87%) of them had data by both parent- and self-report.

### Assessment of the UK sample

Each patient was assessed by a Specialty Registrar in Child and Adolescent psychiatry and diagnoses were reviewed in multidisciplinary team meetings led by a senior psychiatrist. All UK participants completed the Strengths and Difficulties Questionnaire (SDQ), but were not assessed by a psychiatric interview. The SDQ is a 25-item questionnaire with robust psychometric properties (Bourdon, Goodman, Rae, Simpson, & Koretz, 2005; Goodman, 2001) that generates dimensional scores for emotional, behavioral, hyperactivity, and peer problems, as well as prosocial behavior. All parents, and children aged 11 and above (52/88), were asked to provide data. To avoid item (i.e., criterion) overlap with the ARI, the temper tantrum item was excluded from the behavioral scale for analyses (so the sum of the rest of the conduct items was used instead).

#### Analyses

All analyses were conducted separately by reporting source (parent- vs. self-report) and sample (US and UK).

For our first aim, the means of items were calculated for each sample. In addition, the single-factor structure of the ARI was tested in a confirmatory factor analysis. This was conducted using the six ARI items in the US and the UK sample. Because of the categorical nature of the items, weighted least square estimation was used as recommended (Yu, 2002). Fit was assessed on the basis of the following fit indices: Comparative Fit index (CFI; 0.95 and above indicates good fit) the Tucker Lewis Index (TLI; values close to 1 indicate good fit) the root mean square error of approximation (RMSEA; values smaller than 0.05 indicate good fit), and the weighted root mean square residual (WRMR; recommended cutoff at 0.6 (Yu, 2002). Internal consistency was estimated using Cronbach’s alpha. Exploratory analyses for longitudinal stability were conducted using Pearson correlation coefficients.

Repeated measures *t*-tests were run in both samples to compare the item means between parent- and self-reported data. The second aim was tested in the US sample using ANOVA comparing parent- or self-reported irritability across healthy volunteers, children at family risk for bipolar disorder, BD, and SMD, with post hoc testing between groups.

In the UK sample, exploratory correlations were run between irritability and scores on the SDQ subscales. To examine our hypothesis that irritability would be associated with emotional problems, rather than conduct or antisocial disorders, a regression model was estimated. In this regression, the outcome variable was the emotional problems scale scores of the SDQ. The total ARI score, as well as the hyperactivity, conduct, peer problems, and prosocial scale scores of the SDQ were entered as predictors all at once. Another regression model was also estimated with the conduct problems scale score of the SDQ as the outcome, and all previously mentioned scale scores were entered all at once as predictors (including the emotional problems scale score). Parent-reported outcomes were predicted by parent-reported variables; self-reported outcomes were predicted by self-reported variables. In addition, association between the total ARI score with the three levels (‘not true’, ‘somewhat true’, ‘certainly true’) of the impairment item (seventh ARI item) was tested using ANOVA in both samples.

#### Ethical approval

US participants were enrolled in an Institutional Review Board approved study at the Intramural Research Program of the National Institute of Mental Health. Parents and children provided written informed consent/assent. The UK study received approval from the East London Ethics Committee (10/H0701/115).

## Results

### Aim 1: Descriptive statistics, internal reliability, factorial structure, longitudinal stability, item comparison between reporters and relationship with age and gender

Item means and standard deviations for the whole sample are shown in Table 1. By either reporting source, being easily annoyed by others was one of the most common items, whereas the two duration items, ‘stay angry for a long time’ and ‘angry most of the time’ were more rare. Total ARI scores by parent- and self-report were higher for the US sample than for the UK sample. In the US sample, Cronbach’s alpha was 0.92 and 0.88 and in the UK sample 0.89 and 0.90, for the parent- and self-report scales, respectively.

Table 1 shows the results of the confirmatory factor analysis: the CFI, TLI and WRMR all suggest that a one-factor solution is an adequate description of the data. Only the RMSEA was higher than the recommended benchmark for the self-reported scales.

We compared, using repeated measures *t*-tests, the item means between parent- and self-report in each sample (Table 2A,B). There are no statistically significant differences for any of the items in the UK sample. However, in the US sample, the overall direction was for higher mean scores by parent-report, with four of six items being statistically significantly higher by parent-report than by self-report.

**Table 2:** (A) *t*-tests for individual items in the US sample. (B) *t*-tests for individual items in the UK sample

	Mean (*SD*) *parent n* = 192	Mean (*SD*) *self n* = 192	*t*-test statistics (*df* = 191)
Easily annoyed by others	0.88 (0.83)	0.88 (0.70)	*t* = 0.00^ ns^
Often lose temper	0.76 (0.84)	0.61 (0.70)	*t* = 2.64^*^^*^
Stay angry for a long time	0.39 (0.59)	0.38 (0.58)	*t* = 0.31^ ns^
Angry most of the time	0.30 (0.60)	0.18 (0.45)	*t* = 2.50^*^^*^
Get angry frequently	0.67 (0.82)	0.48 (0.69)	*t* = 3.03^*^^*^
Lose temper easily	0.79 (0.89)	0.61 (0.79)	*t* = 3.03^*^^*^

	Mean (*SD*) *parent n* = 45	Mean (*SD*) *self n* = 45	*t*-test statistics (*df* = 44)
Easily annoyed by others	0.82 (0.78)	0.73 (0.69)	*t* = 1.07^ns^
Often lose temper	0.51 (0.76)	0.62 (0.75)	*t* = −1.40^ns^
Stay angry for a long time	0.38 (0.65)	0.49 (0.73)	*t* = −1.04^ns^
Angry most of the time	0.17 (0.44)	0.31 (0.60)	*t* = −1.63^ns^
Get angry frequently	0.44 (0.69)	0.47 (0.69)	*t* = −0.22^ns^
Lose temper easily	0.56 (0.69)	0.64 (0.77)	*t* = −1.07^ns^

Parent- and self-report scales were strongly and significantly correlated: *r* = 0.58 (CI 0.47–0.66) and *r* = 0.73 (CI 0.56–0.85) for US and UK samples, respectively. In the US sample, there was no relationship between age and either parent- (*r* = −0.06, CI −0.19 to 0.08) or self-report (−0.10, CI −0.24 to 0.04) total score. In the UK sample, there was a relationship between age and parent- (*r* = −0.26, CI −0.45 to −0.27) but not self-report (*r* = −0.12, CI −0.38 to 0.17) and ARI total score.

In the US sample, by parent-report, there was no difference in irritability levels between boys (3.66, *SD* = 3.74) and girls (3.59, SD = 4.12), as assessed by t-test (*t* = 0.12, *df* = 212, *p* = 0.91). There was also no difference by self-report between boys (3.17, *SD* = 0.62) and girls (3.09, *SD* = 3.18), (*t* = 0.17, *df* = 192, *p* = 0.86).

In the UK sample, by parent-report, there was no difference in irritability levels between boys (3.51, *SD* = 3.48) and girls (parent-report: 3.07, *SD* = 3.29), (*t* = 0.58, *df* = 81, *p* = 0.56). Also, by self-report, there were no differences between boys (2.48, *SD* = 0.62) and girls (4.16, *SD* = 3.69), (t = 1.75, *df* = 48, *p* = 0.09).

The correlation coefficient for the longitudinal stability (over an average of about 1 year) was high and significant by parent- (*r* = 0.88, *p* < 0.001), but not by self- report (*r* = 0.29, *p* = 0.28).

### Aim 2: Validation of scale

#### Differences between healthy volunteers, children at family risk for BD, children with BD, and children with SMD in the US sample

**Figure 1:**
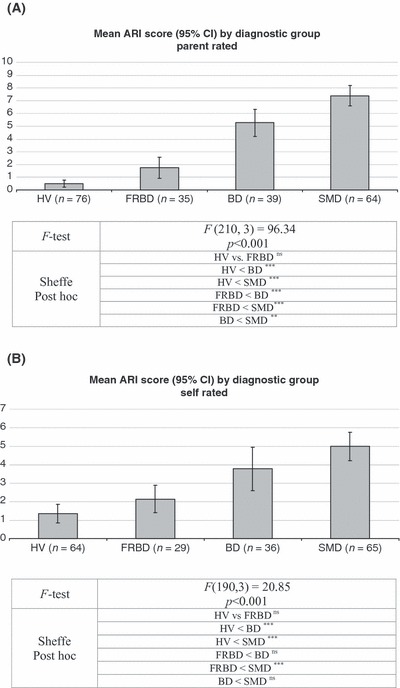
(A) Mean ARI score (95% CI) by diagnostic group parent-rated. ‘ns’ denotes no statistically significant differences, sample sizes reflect those with parent-report ARI data available.***p < 0.001, **p < 0.01. HV, healthy volunteers. FRBD, children at family risk for bipolar disorder. BD, children with bipolar disorder. SMD, severe mood dysregulation. (B) Mean ARI score (95% CI) by diagnostic group self-rated. ‘ns’ denotes no statistically significant differences, sample sizes reflect those with self-report ARI data available.***p < 0.001, **p < 0.01. HV, healthy volunteers. FRBD, children at family risk for bipolar disorder. BD, children with bipolar disorder. SMD, severe mood dysregulation

Figure 1A,B illustrates the gradation in irritability with lowest scores in healthy volunteers and highest scores in SMD, by either reporting source. By parent-report, all individual comparisons were significantly different, with the exception of that between healthy volunteers and children at family risk for BD. In particular, SMD showed significantly more irritability than BD. By self-report, SMD and BD, but not children at family risk for BD, showed significantly more irritability than healthy volunteers. SMD also reported more irritability than children at family risk for BD. The difference between SMD and BD was not significant by self-report.

#### Differential association of irritability

Exploratory univariate correlation analyses indicated that the ARI correlated with all SDQ subscales, with the exception of self-reported peer problems (Table 3). In the univariate analyses, the association between irritability and emotional problems was comparable to the association between irritability and conduct problems by either reporting source: the confidence intervals of the correlation coefficients between ARI total score and emotional problems overlapped with the confidence intervals of the correlation coefficients between ARI total score and conduct problems (Table 3).

**Table 3:** Pearson correlations (and 95% confidence intervals) between ARI and other SDQ scores. Parent-report is above and self-report below the diagonal (UK sample)

	Irritability (ARI)	Emotional (SDQ)	Conduct (SDQ)	Hyperactivity (SDQ)	Peer (SDQ)	Prosocial (SDQ)
Irritability (ARI)	–	0.60 (0.39 to 0.75)	0.55 (0.31 to 0.72)	0.33 (0.05 to 0.56)	0.23 (−0.06 to 0.49)	−0.55 (−0.72 to −0.31)
Emotional (SDQ)	0.66 (0.52 to 0.77)	–	0.36 (0.08 to 0.58)	0.48 (0.23 to 0.67)	0.28 (−0.00 to 0.53)	−0.37 (−0.59 to −0.09)
Conduct (SDQ)	0.48 (0.29 to 0.63)	0.43 (0.24 to 0.59)	–	0.29 (0.00 to 0.53)	0.32 (0.04 to 0.55)	−0.21^ns^(−0.46 to 0.08)
Hyperactivity (SDQ)	0.39 (0.19 to 0.56)	0.38 (0.18 to 0.55)	0.49 (0.30 to 0.64)	–	0.04^ ns^(−0.24 to 0.32)	−0.04^ns^(−0.32 to 0.25)
Peer (SDQ)	0.30 (0.09 to 0.48)	0.38 (0.18 to 0.55)	0.28 (0.07 to 0.47)	0.25 (0.03 to 0.44)	–	−0.06^ns^(−0.22 to 0.34)
Prosocial (SDQ)	−0.57 (−0.67 to −0.36)	−0.24 (−0.43 to −0.02)	−0.21^ns^(−0.41 to 0.00)	−0.13 (−0.33 to 0.09)	0.03 (−0.24 to 0.19)	–

ARI, Affective Reactivity Index; SDQ, Strengths and Difficulties Questionnaire.

Parent-report *n* = 82 (one subject with ARI but no SDQ data); self-report: *n* = 48 (two subjects with ARI but no SDQ data).

However, multivariate regression models show that, by parent-report, irritability was the sole predictor of emotional problems (Table 4). By contrast, hyperactivity was the sole predictor of conduct problems (Table 4). By self-report, both irritability and hyperactivity predicted emotional problems and only irritability predicted conduct problems. Note that parent-reported outcomes were predicted by parent-reported variables and self-reported outcomes were predicted by self-reported variables.

**Table 4:** Association between irritability, conduct, and emotion problems in the UK sample

	Outcomes			
	Parent *n* = 81		Self *n* = 48	
Predictors	Emotional (SDQ)	Conduct (SDQ)	Emotional (SDQ)	Conduct (SDQ)
Irritability (ARI)	0.61^*^^*^^*^ (0.38 to 0.84)	0.35 (−0.07 to 0.78)	0.36^*^ (0.04 to 0.68)	0.63^*^^*^ (0.18 to 1.07)
Hyperactivity (SDQ)	0.10 (−0.10 to 0.30)	0.50^*^^*^ (0.20 to 0.81)	0.36^*^^*^ (0.11 to 0.60)	0.18 (−0.20 to 0.55)
Conduct (SDQ)	0.06 (−0.08 to 0.20)	n/a	−0.04 (−.26 to 0.18)	n/a
Emotional (SDQ)	n/a	0.15 (−022 to 0.52)	n/a	−0.08 (−052 to 0.37)
Peer (SDQ)	0.15 (−0.03 to 0.33)	0.09 (−0.20 to 0.38)	0.21 (−0.03 to 0.45)	0.27 (−0.08 to 0.61)
Prosocial (SDQ)	0.11 (−0.08 to 0.31)	−0.02 (−0.34 to 0.30)	−0.18 (−0.45 to 0.10)	0.04 (−0.36 to 0.45)

ARI, Affective Reactivity Index; SDQ, Strengths and Difficulties Questionnaire.

Beta coefficients (and confidence intervals) are reported from linear regression models; ^*^^*^^*^p < 0.001, ^*^^*^p < 0.01, ^*^p < 0.05. Parent-reported outcomes were predicted by parent-reported variables and self-reported outcomes were predicted by self-reported variables.

### Associations between irritability symptoms and impairment due to irritability

By either reporting source, increases in reported impairment were associated with significantly increased irritability in both samples, with the exception of the difference between the intermediate (a little) and top (a lot) category of impairment by self-report, which was not significant (Appendix S1).

## Discussion

This article reports on the characteristics of the ARI, a concise parent- and self-reported questionnaire designed to assess youth irritability.

Our first aim was to describe the basic characteristics of the scale in the US and UK samples. We found a similar pattern of item frequencies across reporting sources and samples, although the absolute frequencies of the items varied. The two items on prolonged anger were endorsed least often. Moreover, we found that while the item means between reporting sources were very similar in the UK sample, they were significantly higher by parent- compared to self-report for most items in the US sample. The higher severity of irritability in the specialized US sample may underlie these differences. The ARI items showed excellent internal consistency and good factorial structure – only by self-report did one of the four indices, the RMSEA, not suggest an optimal fit. These results demonstrate the utility of the ARI across clinic and community-based samples in two countries and across informants, suggesting that the scale can be used transnationally. The longitudinal stability of the ARI seems promising (at least by parent-report), although this inference was drawn using a very small subsample and will require replication.

Our second aim was to examine how the scale is associated with psychopathology. In the US sample, by parent-report, irritability was highest in SMD compared with healthy volunteers, children at family risk for bipolar disorder, and BD. However, irritability in BD was higher than in healthy volunteers or in children at family risk for BD. By self-report, however, differences in irritability between SMD and BD were non-significant, indicating that youth-report is less good at differentiating between these phenotypes. These results highlight the fact that a high level of chronic irritability, while a defining feature of SMD (Leibenluft et al., 2003), may also occur in children with BD. It should also be noted that the BD phenotype examined here is designed to be narrow, that is, to only include children with elated or expansive mood, who may or may not also have irritability, but not the rare group of children (Hunt et al., 2009) presenting with episodic irritability only.

In keeping with our hypothesis (Stringaris & Goodman, 2009a,b), parent-reported irritability was the only predictor of emotional problems when compared with all other SDQ subscales in multivariate models; conversely, only hyperactivity, but not irritability, predicted conduct problems. However, by self-report, irritability and self-reported hyperactivity problems predicted emotional problems, and self-reported irritability also strongly predicted self-reported conduct problems. It should also be noted that in the univariate analyses, irritability was related to either emotional or conduct problems, by either reporting source. This suggests that irritability in this age group acts as an indicator of either conduct or emotional problems. As previously suggested (Stringaris, Zavos, Leibenluft, Maughan, & Eley, 2011), this relationship between irritability and conduct problems may itself be mediated through headstrong and hurtful behaviors, which were not controlled here. A related, but not identical, dimension of negative affect has been recently identified as part of oppositional problems (Burke, Hipwell, & Loeber, 2010).

This study’s strengths include the use of samples across two countries spanning a number of diagnoses and ascertainment methods, and the comparison between SMD and BD. However, it also has a number of weaknesses. First, the samples are subject to referral and Berkson bias, limitations common to all clinic-based studies (Caron & Rutter, 1991). To address this, the ARI should be validated in epidemiologic samples. Second, the size of the UK sample is small. Future studies with larger numbers should be conducted to examine how irritability predicts, for example, treatment outcomes in clinics. Third, the cross-sectional nature of our main results limits the inferences that can be drawn. Fourth, this scale was not designed as an in-depth probe of the phenomenology of irritability or of its relationship with phenotypes of more general emotional and behavioral dyscontrol (Holtmann et al., 2010). Further research is needed to understand these relationships. Also, the ARI was developed to capture irritability in a way that would be accessible to participants from as wide a child age-range as possible. Future research should determine whether it could also be used for adult self-report of irritability.

In conclusion, the ARI demonstrates promising psychometric properties and it may prove a useful tool for clinical and research purposes. Future epidemiologic samples and clinic samples with treatment designs using the ARI may further assess the importance of irritability to psychopathology.

**Key points**

- Irritability is a form of mood dysregulation of intense scientific interest.
- The Affective Reactivity Index is a concise (seven item) scale for the dimensional measurement of irritability.
- The ARI has excellent internal consistency and forms a single factor in both parent- and self-report forms.
- The parent- and self-reported ARI total score differentiates cases from controls in a clinic and a community sample. The parent-rated ARI total score also differentiates between youth with severe mood dysregulation and youth with bipolar disorder.
- The ARI may be a useful tool for the measurement of irritability.

## Footnotes

1 The scale’s initials are an anagram of ‘ira’, which is Latin for anger and rage.

## Supporting information

**Appendix S1:** The Affective Reactivity Index
