# Supplementary material for: The Affective Reactivity Index: a concise irritability scale for clinical and research settings
Source: J Child Psychol Psychiatry. 2012 Nov;53(11):1109–17. doi: 10.1111/j.1469-7610.2012.02561.x (PMC3484687; doi:10.1111/j.1469-7610.2012.02561.x)
Supplement: Supplementary file 1 [file jcpp0053-1109-SD1.doc]

Appendix to Stringaris et al, *The Affective Reactivity Index*

**METHODS**

BD CRITERIA

BD subjects met DSM-IV criteria for BDI/II, with a history of at least one clearly demarcated hypomanic or manic episode (lasting >4 days for hypomania or >7 days for mania) characterized by abnormally elevated or expansive (but not just irritable-only) mood accompanied by three of the “B” criteria. Participants with SMD were required to meet criteria indicating severe irritability and symptoms of hyperarousal (Leibenluft et al., 2003).

SMD CRITERIA

Participants with SMD fulfilled the following criteria: 1) abnormal mood (anger or sadness), present at least half of the day most days, noticeable by people in the child’s environment (e.g., parents, teachers, peers); 2) increased reactivity to negative emotional stimuli (e.g., temper outbursts inappropriate for age or precipitating event) at least three times weekly; 3) hyperarousal symptoms (i.e., at least three: insomnia, intrusiveness, pressured speech, flight of ideas/racing thoughts, distractibility, psychomotor agitation); 4) symptom onset before age 12 years; 5) symptoms present for at least 1 year, with no remission period >2 months; and 6) severe impairment in at least one setting (i.e., home, school, peers), and at least mild impairment in another. Euphoric mood or distinct hypomanic or manic episodes lasting >1 day were exclusionary (Leibenluft et al., 2003).

AT RISK YOUTH

At risk youth (FRBD) had at least one first-degree relative with BD. At risk youth with ADHD or anxiety disorders were included, although a history of major depressive episode or other mood disorder was exclusionary. Healthy volunteers (HV) had no personal history of psychiatric illness, and no first-degree relative with a mood disorder. Exclusion criteria for all groups included IQ <70, pervasive developmental disorder, unstable medical illness, or substance abuse within the past 2 months.

**RESULTS**

Supplementary **Table 1.** Comorbid diagnoses in the US sample

|  | FRBD  (33) | BD  (38) | SMD  (65) |
| --- | --- | --- | --- |
|  | % (n) | | |
| MDD | 3.0 (1) | 44.7 (17) | 21.5 (14) |
| Psychosis | 0.0 (0) | 7.9 (3) | 0.0 (0) |
| Adjustment | 0.0 (0) | 2.6 (1) | 3.1 (2) |
| BP I | 0.0 (0) | 86.8 (33) | n/a |
| BP II | 0.0 (0) | 15.8 (6) | n/a |
| Panic | 0.0 (0) | 13.2 (5) | 0.0 (0) |
| SAD | 3.0 (1) | 36.8 (14) | 40.0 (26) |
| SpPh | 0.0 (0) | 44.7 (17) | 26.2 (17) |
| SoPh | 6.1 (2) | 28.9 (11) | 18.5 (12) |
| GAD | 9.1 (3) | 63.2 (24) | 44.6 (29) |
| OCD | 0.0 (0) | 5.3 (2) | 6.2 (4) |
| PTSD | 0.0 (0) | 2.6 (1) | 1.5 (1) |
| Eliminating | 12.1 (4) | 36.8 (14) | 23.1 (15.65) |
| Eating | 0.0 (0) | 0.0 (0) | 0.0 (0) |
| ADHD | 12.1 (4) | 84.2 (32) | 84.6 (55) |
| CD | 0 (0) | 5.3 (2) | 0.0 (0) |
| ODD | 6.1 (2) | 42.1 (16) | 83.1 (54) |
| Tic | 0.0 (0) | 18.4 (7) | 10.8 (7) |
| PDD | 0.0 (0) | 10.5 (4) | 4.6 (3) |
|  |  |  |  |

SAD=Separation Anxiety Disorder, SpPh= Specific Phobia, SoPh=Social Phobia, GAD= Generalised Anxiety Disorder, OCD= Oppositional Defiant Disorder; PTSD=posttraumatic stress disorder, ADHD=Attention Deficit Hyperactivity Disorder, CD=Conduct Disorder, ODD=Oppositional Defiant Disorder; PDD=Pervasive Developmental Disorder

Note: missing data on 1 subject with BD and ARBD and 2 subjects with SMD

Supplementary Table 2. **Mood states in the US sample**

|  | FRBD  (n=35) | BD  (n=37) | SMD  (n=63) |
| --- | --- | --- | --- |
|  | % (n) | | |
| Euthymic | 100 (35) | 65.8 (25) | 96.8 (61) |
| Hypomanic | n/a | 28.9 (11) | n/a |
| Depressed | n/a | 2.6 (1) | 3.2 (2) |
| Mixed | n/a | 2.6 (1) | n/a |

Note: missing data on 1 subject with BD and 4 subjects with SMD.

**Supplementary Table 3: Gender and age composition of clinic and community samples**

|  | ***Clinic*** | ***Community*** | *statistics* |  |
| --- | --- | --- | --- | --- |
|  |  |  |  |  |
|  | ***N (%)*** | ***N (%)*** |  |  |
| Total | 34 (39%) | 54 (61%) |  |  |
| Boys | 22 (65%) | 30 (56%) |  |  |
| Girls | 12 (35%) | 24 (44%) | *p<0.05; x^2^*=0.723 | |
|  |  |  |  |  |
|  | **mean (SD)** | **mean (SD)** |  |  |
|  | 11.85(SD=3.611) | 11.59(SD=3.401) |  |  |
| Age |  |  | p=0.734; t= 0.341, df=86 | |
|  |  |  |  |  |

**Supplementary Table 4: Diagnostic and clinical characteristics of the UK clinic sample (n=34)**

| **Clinical presentation** | | **Number (%)** |
| --- | --- | --- |
| Attention Deficit Hyperactivity Disorder (ADHD) | | 5 (14.7%) |
| Oppositional Defiant Disorder/ Conduct Disorder (ODD/CD) | | 5 (14.7%) |
| Anxiety disorder | | 5 (14.7%) |
| Autism Spectrum Disorder (ASD) | | 3 (8.8%) |
| Self harm |  | 3 (8.8%) |
| Depression | | 1 (2.9%) |
| Depression with self harm | | 3 (8.8%) |
| ADHD with ODD/CD | | 1 (2.9%) |
| ADHD with Mild learning disability | | 1 (2.9%) |
| Tics | | 1 (2.9%) |
| Eating disorder with self harm | | 1 (2.9%) |
| Psychosis with Mild learning disability | | 1 (2.9%) |
| No diagnosis | | 4 (11.8%) |

Supplementary **Table 5**: Strengths and Difficulties Questionnaire (SDQ) scores in the UK sample.

|  |  | **SDQ total** | **Emotional** | **Conduct** | **Hyperactive** | **Peer problems** | **Prosocial** |
| --- | --- | --- | --- | --- | --- | --- | --- |
|  |  | **Possible range**  **0-40** | **Possible range**  **0-10** | **Possible range**  **0-10** | **Possible range**  **0-10** | **Possible range**  **0-10** | **Possible range**  **10-0** |
| Parent | Clinic  (n=53) | 19.43 (6.548) | 5.03 (2.798) | 4.43 (3.148) | 6.23 (2.582) | 3.73 (2.067) | 6.23 (1.755) |
|  | Community  (n=30) | 5.68 (3.518) | 1.28 (1.378) | 0.96 (1.109) | 2.47 (2.006) | 0.96 (0.999) | 8.23 (1.739) |
|  | t (df) | 12.473 (81)* | 8.186 (81)* | 7.295 (81)* | 7.385 (81)* | 8.233 (81)* | (-)4.999 (81)* |
|  |  |  |  |  |  |  |  |
| Self | Clinic  (n=29) | 19.89 (4.677) | 5.84 (2.911) | 4.00 (1.732) | 6.21 (2.200) | 3.84 (2.243) | 6.05 (1.840) |
|  | Community  (n=29) | 7.79 (6.103) | 2.45 (2.473) | 1.34 (1.446 | 2.48 (1.724) | 1.52 (1.724) | 8.52 (1.353) |
|  | t (df) | 7.337 (46)* | 4.335 (46)* | 5.751 (46)* | 6.562 (46)* | 4.053 (46)* | (-)5.347 (46)* |
|  |  |  |  |  |  |  |  |
| Means and standard deviations are presented with t-values and degrees of freedom for each comparison *p<0.001 | | | | | | | |

*Relationship between ARI total score and the ARI impairment question (item 7)*

**US Sample:**

Parent Report (Mean, SD)

*no*: 0.81, 1.5

*a little*: 2.79, 2.63;

*a lot*: 8.19, 2.55;

*Overall Anova*:

F=241.45 (df=2), p<0.001)

*Post hoc Sheffe test*:

no<a little (p<0.001)

no<a lot (p<0.001)

a little< a lot (p<0.001)

**US Sample:**

Self Report (Mean, SD)

*no*: 1.13, 1.73

*a little*: 4.0, 2.39

*a lot*: 7.46, 2.63

*Overall Anova*:

F=103.15 (df=2), p<0.001)

*Post hoc Sheffe test:*

no<a little (p<0.001)

no<a lot (p<0.001)

a little< a lot (p<0.001)

**UK Sample**

Parent Report (Mean, SD)

*no*: 1.00, 1.37

*a little*: 5.29, 2.37

*a lot*: 7.37, 2.77;

*Overall Anova*:

F=80.34 (df=2), p<0.001)

*Post hoc Sheffe test:*

no<a little (p<0.001)

no<a lot (p<0.001)

a little< a lot (p<0.01)

**Self report (Mean, SD)**

*no*: 1.00, 1.77

*a little*: 6.31, 2.90

*a lot*: 6.86, 2.19

*Overall Anova*:

F=37.99 (df=2), p<0.001)

*Post hoc Sheffe test:*

no<a little (p<0.001)

no<a lot (p<0.001)

a little< a lot (p>0.05) not significant

LEIBENLUFT, E., CHARNEY, D. S., TOWBIN, K. E., BHANGOO, R. K. & PINE, D. S. (2003). Defining clinical phenotypes of juvenile mania. *American Journal of Psychiatry,* 160**,** 430.
